# CT Imaging Manifestations of Tuberculous Aortic Aneurysm

**DOI:** 10.31083/j.rcm2308271

**Published:** 2022-07-26

**Authors:** Xiaona Xing, Zhonghua Sun, Li Chen, Nan Zhang, Wei Xiong, Yu Li

**Affiliations:** ^1^Department of Neurology, The Third Affiliated Hospital of Shenzhen University, Shenzhen Luohu People’s Hospital, 518000 Shenzhen, Guangdong, China; ^2^Discipline of Medical Radiation Science, Curtin Medical School, Curtin University, Perth, WA 6102, Australia; ^3^Department of Radiology, Beijing Anzhen Hospital, Capital Medical University, 100029 Beijing, China; ^4^Department of Respiration, First Teaching Hospital of Tianjin University of Traditional Chinese Medicine, 300380 Tianjin, China; ^5^Department of Radiology, The Seventh Affiliated Hospital of Sun Yat-sen University, 518107 Shenzhen, Guangdong, China

**Keywords:** tuberculosis, tuberculous aortic aneurysm, computed tomography, imaging, diagnosis

## Abstract

**Background:**

Tuberculous aortic aneurysm (TBAA) is a rare complication of TB and is associated with high mortality. Early diagnosis is critical; however, it is challenging due to nonspecific symptoms. This study summarized the computed tomography (CT) features of TBAA with the aim of assisting with timely clinical diagnosis.

**Methods:**

Seventeen patients with TBAA between 2015 and 2020 were included in this study. The clinical manifestations, past medical history, laboratory and imaging examinations, treatments, and other data were collected and analyzed. CT angiography was performed in all patients.

**Results:**

All tuberculous aneurysms were pseudoaneurysms, which were located in the thoracic aorta (8/17, 47%), abdominal aorta (7/17, 41%), junction of the thoracic and abdominal aorta (1/17, 6%) or abdominal aorta and iliac artery (1/17, 6%) region. The shapes of all aneurysms were saccular, and nine of them were lobulated. The aneurysm diameter ranged from 3 to 12 cm. Of the 17 patients, 12 (71%) had calcification; 14 (82%) had intraluminal thrombus; 12 (71%) showed enlarged lymph nodes, which were closely related to the aneurysm; and 9 (53%) had tuberculous spondylitis including TB of the thoracic lumbar and lumbosacral spine. Psoas abscess was detected in 4 (23%) patients and iliopsoas abscess was detected in 1 (6%) patient.

**Conclusions:**

TBAA typically shows mycotic shapes on CT scans. Another feature is that the surrounding tissues and adjacent organs of tubercular aneurysms are usually infected with TB, and most of them are accompanied by other sites of TB.

## 1. Introduction

Tuberculosis (TB) is a communicable disease that is a major cause of ill health and the leading infectious disease killer globally [1, 2]. China is one of the high TB burden countries; new TB patients in China accounted for about 8.4% of the world’s cases in 2019, ranking third worldwide [1]. The TB incidence rate has slowly declined since the beginning of the 21st century because of human immunodeficiency virus (HIV) infection, anti-TB drug resistance, and the use of immunosuppressive drugs [2]. Other risk factors for TB are diabetes mellitus, silicosis, smoking, air pollution, malnutrition and protein imbalance [3]. TB typically affects the lungs (pulmonary TB) but can also affect other sites. Tuberculous aortic aneurysm (TBAA) is exceedingly rare [4]. The first case of TBAA was reported by Kamen in 1895 [5, 6]. TBAA is associated with high mortality due to its high risk of sudden rupture. No TBAA patients were known to have survived until the availability of combined technologies of modern imaging, anti-TB therapy, and surgical treatment [5]. Early diagnosis and prompt treatment are important for the improvement of survival [7]. However, early diagnosis is difficult due to the non-specific symptoms and negative blood cultures. Some cases are diagnosed at an advanced stage or after developing complications, such as rupture or aortic fistula.

Current imaging modalities can detect infected aneurysms in clinically suspicious patients including computed tomography (CT), magnetic resonance imaging (MRI), and 18F-fluorodeoxyglucose (FDG) positron emission tomography (PET)/CT [8]. CT angiography (CTA) with arterial and venous phases imaging enables evaluation of the entire aorta. CTA is non-invasive and efficient, with broad coverage and isotropic voxel capabilities; thus, it is increasingly used for the diagnostic assessment of aortic disease including TBAA.

This study retrospectively reviewed 17 cases of patients with TBAA, and collected their clinical data, laboratory tests, imaging findings, and treatment for analysis. The CTA imaging features of all TBAA patients were summarized and analyzed.

## 2. Materials and Methods

### 2.1 Study Participants

This was a multicenter, retrospective study of patients presenting with TBAA at Beijing Anzhen Hospital, Fuyang Hospital of Anhui Medical University, and Liuzhou People’s Hospital from January 2015 to December 2020. A total of 17 cases diagnosed with TBAA were retrieved from the medical records of each clinical center. The diagnosis of TBAA was based on a combination of the following criteria: CTA suggesting infectious AA; accompanied by TB; exclusion of other infectious aneurysms; and standard anti-TB therapy was effective. The clinical manifestations, past medical history, laboratory and imaging examinations, therapies, and other data of patients were collected from the medical record system.

### 2.2 CTA

All of the patients received CTA. CT scans were conducted on a 64- or 128-slice scanner (as the patients were enrolled from several medical centers, the scanners were different). Aortic CTA images were acquired when 75–85 mL contrast medium was administered intravenously at a rate of 4–5 mL/s, followed by intravenous injection of 30–40 mL saline chaser at the same rate as the contrast medium. Images were reconstructed with a slice thickness of 0.5–0.75 mm and a reconstruction interval of 0.25–0.5 mm. Postprocessing of the images (we obtained the original CT (A) Digital Imaging and Communications in Medicine data from all of the centers) was performed on a separate workstation (Vitrea FX Workstationl Vital Images, Minnetonka, MN, USA). Then 1-mm-thick axial, multiplanar reformations, volume-rendered, and maximum intensity projection images of the aorta were produced.

### 2.3 Image Analysis

The CT images were independently reviewed by two cardiothoracic radiologists (with 20 years and 8 years of experience in the field), and final decisions were reached by consensus. As previously reported [7], the following items were regarded as being predictive for mycotic aneurysms on CT images: site of mycotic aneurysms, calcification of the aneurysm wall, absence or presence of aneurysm wall enhancement, presence of air bubble around the aneurysm, bone destruction of adjacent vertebra, soft tissue involvement around the aneurysm (psoas abscess, retroperitoneal abscess, or peritoneal abscess), enlarged lymph nodes near lesions, and other infectious foci outside the aorta.

## 3. Results

### 3.1 Study Population Characteristics

In this study, 12 of 17 (71%) patients with active pulmonary TB included those with bacteriologically positive sputum (smear-positive), with imaging findings of active pulmonary disease. The remaining five patients with extrapulmonary TB included those with typical imaging findings of extrapulmonary TB, in whom anti-TB therapy was effective, known as clinically diagnosed TB.

The clinical and laboratory features and outcomes of the 17 cases are described in Table 1. The mean age of the patients was 57.41 ± 21.27 years old (range, 6–81 years), with a marked male predominance (13 men, 4 women). The main presenting symptoms were fever (7/17, 41%), chest pain (6/17, 35%), abdominal pain (4/17, 24%), low back pain (2/17, 12%), lower limb pain (2/17, 12%), hemoptysis (1/17, 6%), chest distress and dyspnea (1/17, 6%). Four (24%) patients had both chest pain and fever, one (6%) had both abdominal pain and fever, one (6%) had both lower limb pain and fever, and only fever was found in one case (6%). Some of the patients had constitutional symptoms such as weakness, fatigue, or weight loss. One (6%) patient had a history of syphilis and HIV infection. Five (29%) patients had a diabetes history. Extravascular TB was found in all patients such as pulmonary TB in 12 (71%); tuberculous spondylitis in 9 (53%); and renal TB, pleural TB, and prostate TB in 1 (6%). Five of them (29%) had both pulmonary TB and tuberculous spondylitis. The tuberculin skin test of all patients was positive. Sputum TB tests were positive in patients with pulmonary TB (12/17, 71%). The concentration of C-reactive protein and the erythrocyte sedimentation rate were elevated in all patients. The blood cultures were negative in all patients. All patients were treated with anti-TB therapy. Two patients (Patient Nos. 2 and 17) had previous surgery for TB of the thoracic vertebrae. Eight (47%) patients underwent surgery for AA: 6 (35%) received endovascular grafting treatment and the remaining 2 (12%) received open surgical repair. All eight patients were followed up; only one (12%) developed endoleak, whereas all of the others had excellent results. Overall, six (35%) patients died. Rupture with massive bleeding occurred in all deaths, with one patient dying perioperatively. None of these six patients were treated surgically.

**Table 1.** **Summary of the clinical characteristics of 17 patients with TBAA**.

Patients	Age (y)/sex	Symptoms	Past medical history	Extravascular tuberculosis	Sputum tuberculosis test	CRP (mg/L)/ESR (mm/h)	Surgical treatment of aortic aneurysm	Outcome
1	50/F	Chest pain	No	Pulmonary TB	+	83/65	Endovascular grafting	Improvement
2	55/M	Chest pain	No	Thoracic vertebra TB	–	62/45	No	Lost to follow-up
3	81/M	Abdominal pain		Lumbar TB; psoas abscess	–	73/51	Endovascular grafting	Endoleak
4	62/M	Low back pain	Diabetes	Lumbar TB; psoas abscess	–	78/55	Endovascular grafting	Improvement
5	76/M	Abdominal pain	Diabetes	Lumbar TB	–	82/38	No	Lost to follow-up
6	73/F	Chest pain and fever	Hypertension	Pulmonary TB; lumbar TB; psoas abscess	+	98/48	No	Death
7	20/M	Low back pain	No	Pulmonary TB; lumbar TB; psoas abscess	+	88/42	No	Death
8	6/M	Chest pain and fever	No	Pulmonary TB	+	68/33	Open surgical repair	Improvement
9	74/M	Fever, back pain and abdominal pain	No	Pulmonary TB; lumbar TB	+	66/41	No	Death
10	42/M	Left lower limb pain	No	Renal TB; pleural TB; psoas abscess	–	82/55	Endovascular grafting	Improvement
11	74/M	Abdominal pain and cough	Diabetes	Pulmonary TB; lumbosacral TB	+	83/45	No	Death
12	72/M	Fever	No	Pulmonary TB	+	74/38	Endovascular grafting	Improvement
13	47/M	Fever, left lower limb pain	HIV, syphilis	Pulmonary TB; iliopsoas abscess	+	103/65	No	Death
14	57/M	Chest distress and dyspnea	Diabetes	Pulmonary TB, prostate TB	+	87/54	Open surgical repair	Improvement
15	84/M	Fever, chest pain, and hemoptysis	Diabetes	Pulmonary TB	+	112/50	No	Death
16	45/F	Fever and chest pain	No	Pulmonary TB	+	92/58	No	Lost to follow-up
17	58/F	AA was found during thoracic spine tuberculosis surgery	No	Pulmonary TB; thoracic vertebra TB	+	96/39	Endovascular grafting	Improvement

TBAA, tuberculous aortic aneurysm; CRP, C-reactive protein; ESR, the erythrocyte sedimentation rate; F, female; HIV, human immunodeficiency virus; M, male; TB, tuberculosis. The normal range of ESR, male, 0–15 mm/h, female, 0–20 mm/h. The normal range of CRP, 0–8 mg/L.

### 3.2 CTA Imaging Findings

**Fig. 1.**
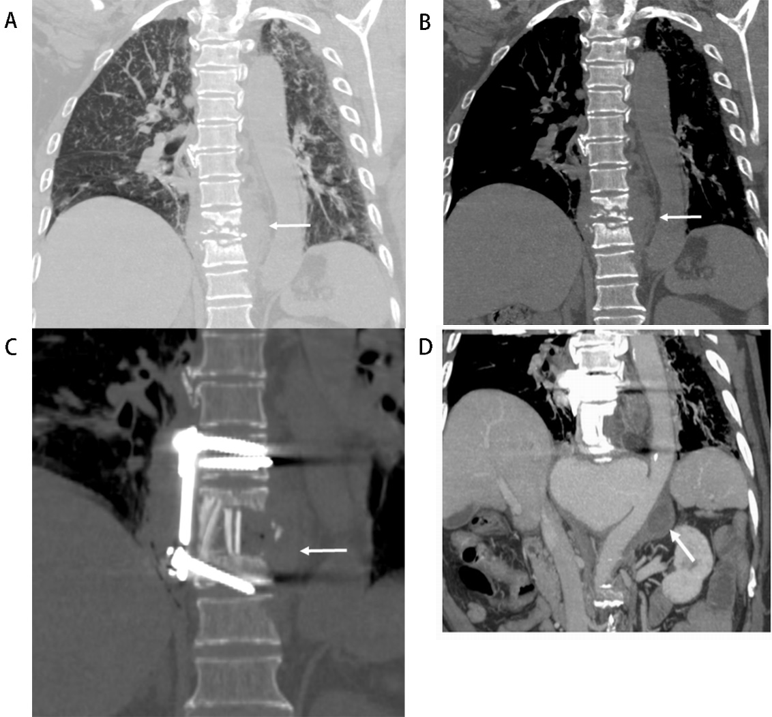
**Tuberculous spondylitis involved the aorta (Patient No. 9)**. A 74-year-old man presented with repeated fever combined with back pain 8 months ago and abdominal pain for 1 month. (A,B) Computed tomography (CT) of lung scan images in September 2018. Multiple miliary nodulas could be in the bilateral lungs, erosion of the vertebral body and endplate at T9 and T10 and the right side of the T11 vertebral body, soft tissue swelling or abscess around the vertebral body (arrow in A). The aorta outline was distinct, the abscess around the vertebral body at T8 to T11 (arrow in B). (C) One month after tuberculous spondylitis surgery at T9 and T10 without standard anti-tuberculosis drug treatment. The lesion in T11 progressed, and the abscess around the vertebral body was enlarged (arrow), even after debridement was performed. (D) Seven months later, the patient developed abdominal pain. Coronal view of contrast-enhanced CT images in May 2019 demonstrated a large pseudoaneurysm at the level of T11 and enlarged lymph nodes with a hypodense center, which indicated necrosis (arrow). T, thoracic vertebra.

**Fig. 2.**
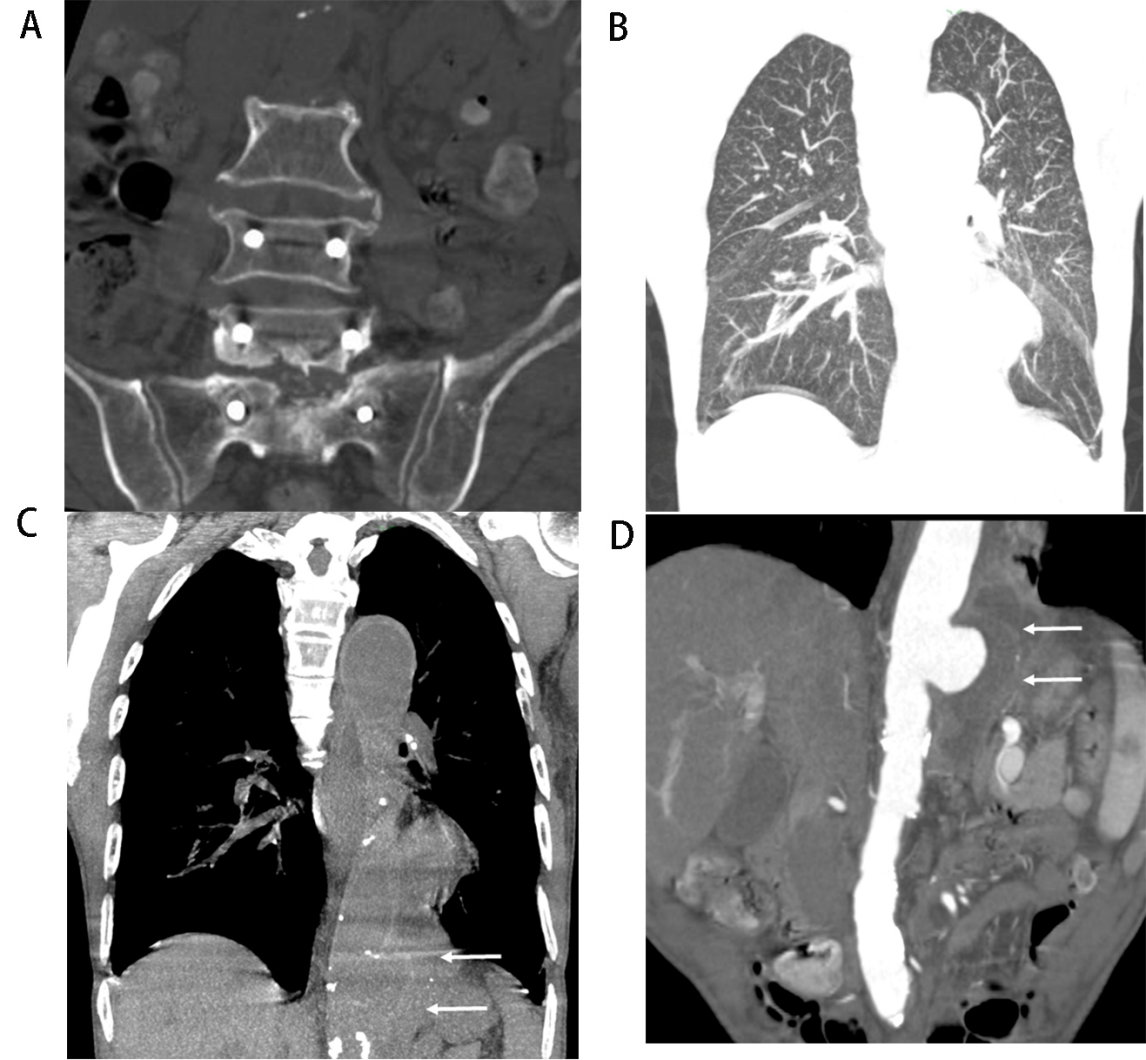
**Tuberculous aortic aneurysm with abscess (Patient No. 11)**. A 74-year-old man, who underwent surgical treatment of lumbosacral TB 1 year prior, complained of cough and abdominal pain for 3 months. (A) Lumbar and sacrum spinal computed tomography (CT) scan showed destruction of the vertebral body from L5 to S1 and internal fixation from L4 to S1. (B) Coronal multiplanar reformation of lung CT demonstrated multiple miliary nodules in bilateral lungs. (C) Outline of the distal segment of the thoracic aorta was obviously enlarged (arrows). (D) Coronal view of abdominal CT angiography showed that the left wall was disrupted and a mural thrombus formed in the lumen (arrows) and lower density in the periaortic soft tissue with septum, which indicated necrosis and abscess. L, lumbar vertebra; S, sacral vertebra.

**Fig. 3.**
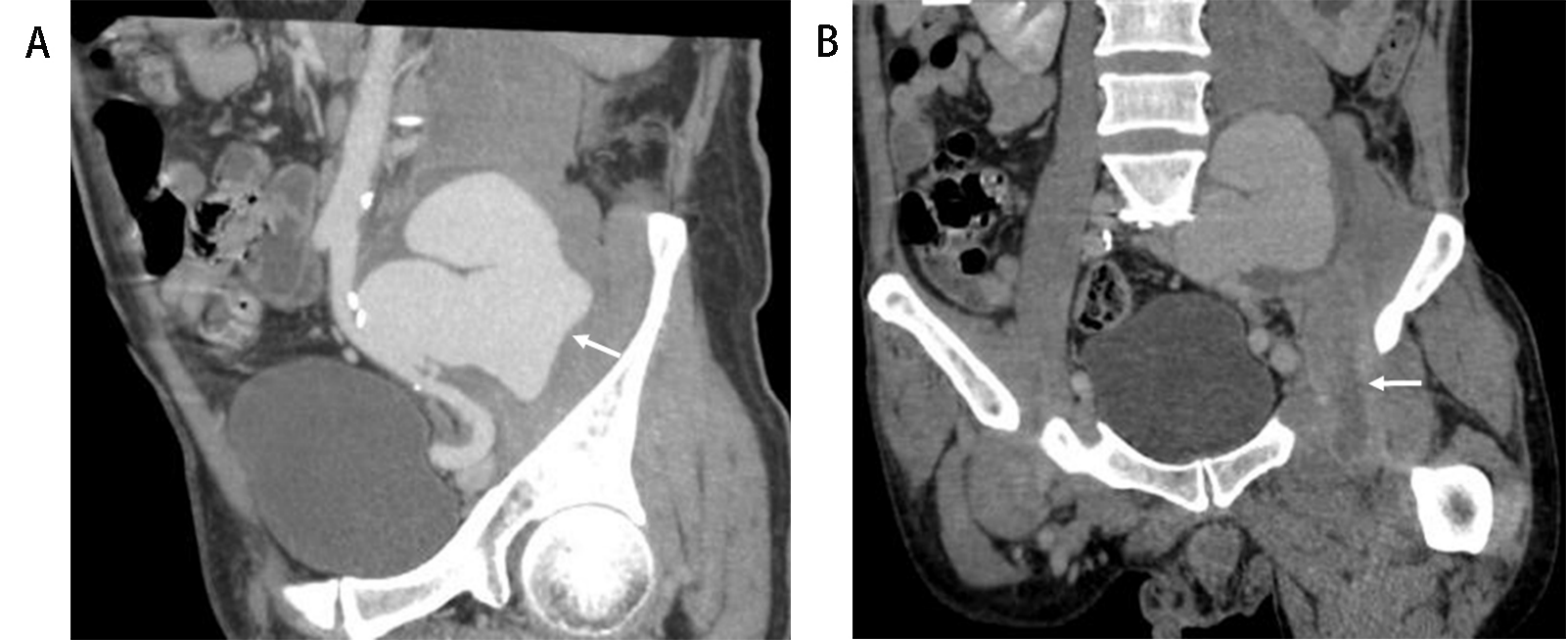
**Iliopsoas abscess associated with tuberculous aortic aneurysm (Patient No. 13)**. A 47-year-old man with a history of human immunodeficiency virus, syphilis, and TB, presented with fever and left lower quadrant abdominal pain for 1 month. (A) Multiple planner reconstruction computed tomography angiography of the left iliac artery showed giant lobulated aortic aneurysm (arrow). (B) Left iliopsoas muscle showed swelling and was enlarged with a relatively low-density area, with contrast-enhanced rim of the abscess wall (arrow).

**Fig. 4.**
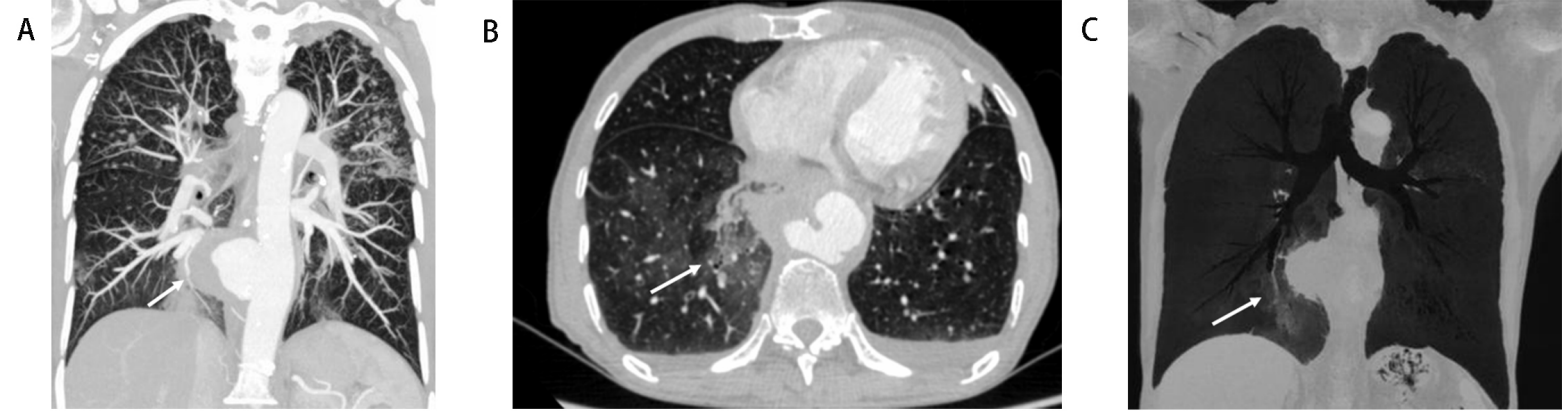
**Aortobronchial fistula (Patient No. 15)**. An 84-year-old male with a history of diabetes mellitus and TB presented with fever, chest pain, and hemoptysis. (A) Maximum-intensity projection image shows multiple small nodules with sharp edges and upper lobe distribution, saccular pseudoaneurysm of the thoracic aorta with surrounding soft tissue (arrow). (B) Transverse view demonstrates patchy ground-glass opacity in the right lower lobe consistent with alveolar hemorrhage (arrow). (C) Minimum-intensity projection image shows the lumen of right basal segmental bronchus (arrow, adjacent to the aneurysm wall) obstruction filled with high-density material.

**Fig. 5.**
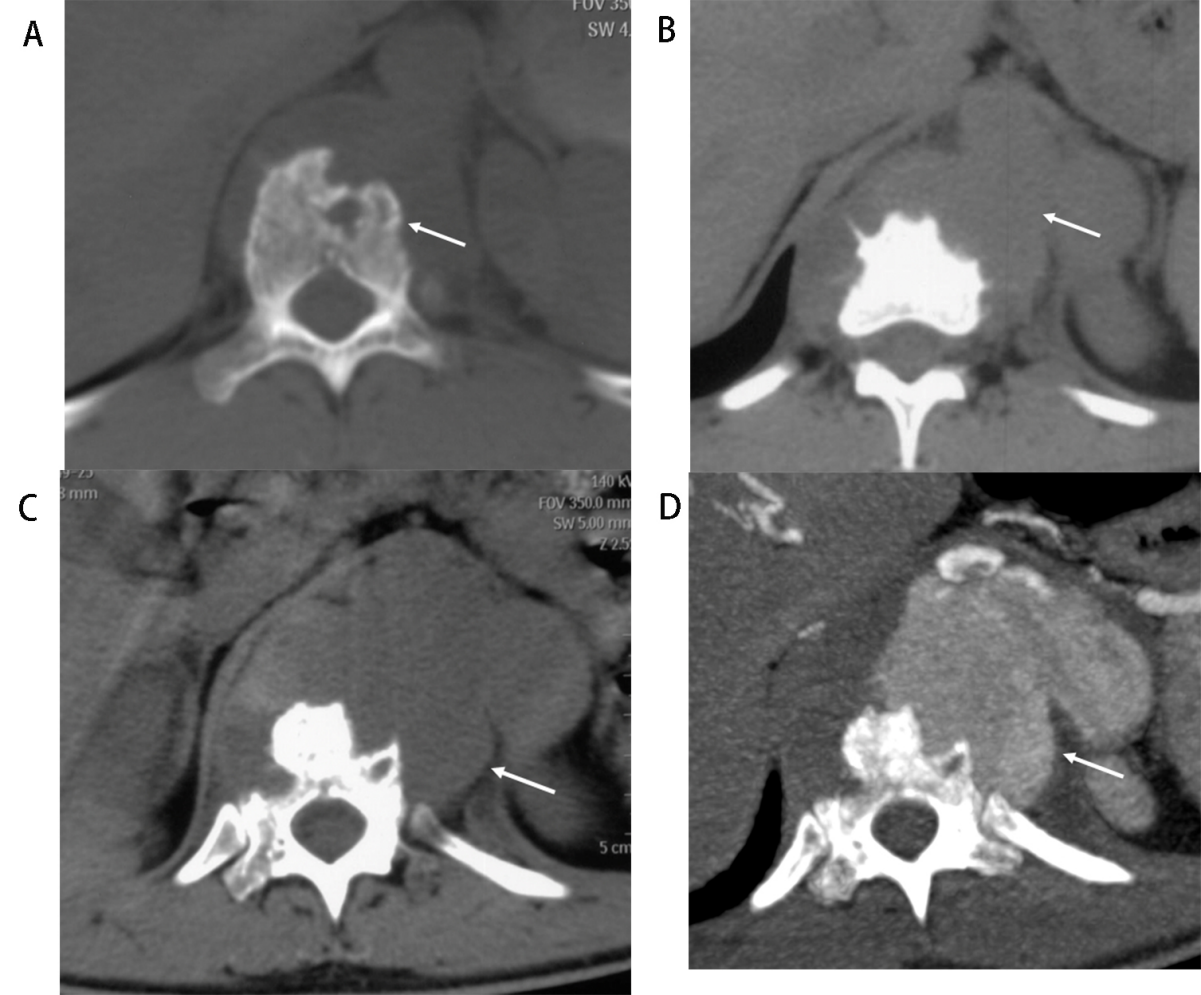
**Rapid growth of TBAA (Patient No. 7)**. A 20-year-old male with a 3-month history of back pain, low fever (37.5–37.9 °C), and a further acute episode of back pain aggravated in recent days. (A) Initial lumbar vertebral computed tomography (CT) imaging (August 20, 2015) shows erosion of the vertebral body and endplate at T11 (arrow) with soft tissue swelling or abscess around the vertebral body. The outline of the aorta is distinct. (B) Lumbar vertebral CT after 53 days (October 13, 2015) reveals obliteration of fat planes between the vertebral body and aorta, the outline of the aorta protrudes to the left posteriorly (arrow). It suggests the aorta is involved. (C) Two weeks later (October 27, 2015), the obvious progression in the vertebral body’s destruction and surround tissue can be shown (arrow), the outline of the aorta is enlarged, which is confirmed by CT angiography (CTA). (D) CTA on November 3 2015 showed lobulated pseudoaneurysm formed adjacent to the eroded vertebral body. T, Thoracic vertebra.

The imaging results of CTA are summarized in Table 2. All tuberculous aneurysms were solitary pseudoaneurysms located in the abdominal aorta (7/17, 41%, Patient Nos. 3, 4, 5, 7, 9, 10, 11; Fig. 1), thoracic aorta (8/17, 47%, Patient Nos. 1, 2, 8, 12, 14, 15, 16, 17; Fig. 2), junction of thoracic and abdominal aorta (1/17, 6%, Patient No. 6), both abdominal aorta and iliac artery (1/17, 6%, Patient No. 13; Fig. 3). The shapes of all aneurysms were saccular, and nine of them were lobulated—the wall of the aneurysm was irregular. In the death group, all six aneurysms were lobulated based on the saccular shape. Four (24%) of the aneurysms were found to have a large saccular appearance with septum (Fig. 2D). The diameter of the aneurysm ranged from 3 to 12 cm. Twelve (71%) patients had calcification, which was consistent with atherosclerosis. Intraluminal thrombus was found in 14 (82%) patients (Fig. 2D). Significant exudation around aneurysm was found in three patients (18%; Fig. 1) and no gas bubbles were found. Necrosis and abscess were found around the aorta (Fig. 2D). Twelve (71%) patients showed enlarged lymph nodes, which were connected to or around the aneurysm (Fig. 1D). Nine (53%) patients had tuberculous spondylitis including TB of the thoracic aorta (2/17, 12%), lumbar and lumbosacral spine (7/17, 41%; Figs. 1,2). Psoas abscess was found in five (29%) patients and iliopsoas abscess (IPA) was found in one patient (6%; Fig. 3). Aortobronchial fistula was detected in one patient (6%; Fig. 4). Multiple miliary nodules were found in bilateral lungs by pulmonary CT (Figs. 1A,2B,4A). Spinal CT scan demonstrated destruction of the vertebral body, and soft tissue swelling or abscess around the vertebral body were also visualized (Figs. 1A,2A,5). The growth of the aneurysm was rapid (Fig. 5).

**Table 2.** **Summary of CT imaging appearances of 17 patients with TBAA**.

Patients	Site	Shape	Outside diameter (mm)	Effusion/gas bubble/Septum	Mural thrombus	Enlarged lymph nodes	Calcification	Surrounding tissue
1	Thoracic aorta	Saccular	39	+/–/ –	–	+	–	Multiple TB nodules in bilateral lung
2	Thoracic aorta	Saccular	36	–/–/–	+	+	+	Destruction of thoracic vertebral body; postoperative thoracic vertebra internal fixation
3	Abdominal aorta	Saccular	110	–/–/–	+	+	+	Destruction of vertebral body from L3 to L5; psoas abscess
4	Abdominal aorta	Lobulated	105	–/–/–	+	+	+	Destruction of lumbar vertebral body; psoas abscess
5	Abdominal aorta	Lobulated	120	–/–/–	+	+	+	Destruction of vertebral body from L3 to L4
6	The junction of thoracic aorta and abdominal aorta	Lobulated	73	–/–/–	–	+	+	Multiple TB nodules in bilateral lung; destruction of lumbar vertebral body; psoas abscess
7	Upper abdominal aorta	Lobulated	83	+/–/+	+	+	–	Multiple TB nodules in bilateral lung; obvious destruction of lumbar vertebral body; psoas abscess
8	Thoracic descending aorta	Saccular and lobulated	30	–/–/–	–	+	–	Multiple TB nodules in bilateral lung
9	Upper abdominal aorta	Large saccular, cyst wall separation	45	+/–/+	+	+	+	Multiple TB nodules in bilateral lung; erosion of the vertebral body and endplate at T9 and T10; perivascular abscess
10	Abdominal aorta	Lobulated	111	–/–/+	+	+	–	Psoas abscess; kidney and pleura involvement
11	Upper abdominal aorta	Saccular and lobulated	100	–/–/–	+	–	+	Multiple TB nodules in bilateral lung; destruction of lumbosacral vertebral body
12	Thoracic aorta	Saccular	32	–/–/–	+	–	+	Multiple TB nodules in bilateral lung
13	Abdominal aorta and iliac artery	Saccular and lobulated	94	–/–/+	+	+	+	Multiple TB nodules in bilateral lung; iliopsoas abscess
14	Ascending aorta	Saccular	59	–/–/–	+	+	+	Multiple TB nodules in bilateral lung
15	Thoracic descending aorta	Saccular and lobulated	72	–/–/–	+	–	+	Multiple TB nodules in bilateral lung; aortobronchial fistula
16	Thoracic descending aorta	Saccular	33	–/–/–	+	–	–	Multiple TB nodules in bilateral lung
17	Descending aortic arch	Saccular	58	–/–/–	+	–	+	Multiple TB nodules in bilateral lung; destruction of vertebral body from T2 to T7

TBAA, tuberculous aortic aneurysm; L, lumbar vertebra; T, thoracic vertebra; TB, Tuberculosis.

## 4. Discussion

Mycotic aortic aneurysm of TB is a rare complication of TB but with high mortality. When mycotic aneurysms are present in the context of TB, and particularly, disseminated TB, TBAA should be suspected [3]. TB of any type was diagnosed on presentation in all of our cases. A few patients had an underlying condition that is known to increase the risk of TB such as HIV infection, diabetes, or oral immunosuppressants [9]. Most of the reported cases of TBAA are symptomatic, but the symptoms are nonspecific and depend on the size, position, and rapid growth of the aneurysm. Patients may describe thoracic, abdominal, or dorsal pain, which may be accompanied by fever. Fever occurs in 35% of patients [3]. In this study group, the proportion of fever was relatively high, with seven patients having fever (41%). They may also present with palpable or a radiographically visible periarterial mass, especially if expanding or pulsatile. Hemorrhage or hypovolemic shock may occur if the aneurysm ruptures or perforates. If a fistula is formed between the aneurysm and the nearby organs, such as the trachea or intestines, massive hemoptysis [10] or gastrointestinal bleeding [11] may occur. The poor prognosis of these patients emphasizes the importance of early diagnosis.

The vast majority of tuberculous aneurysms are pseudoaneurysms (87%), although true (9%) or dissecting (4%) aneurysms have been described [12]. All tuberculous aneurysms of our patients were pseudoaneurysms. About 75% of TBAAs present as a contiguous lesion on the surrounding tissue, such as tuberculous lymphadenitis, pericarditis, empyema, spondylitis, or paravertebral abscess [3]. Caseous necrosis invading the entire arterial wall results in perforation, some with massive hemorrhage or perivascular hematoma formation. Fibrosis gradually forms in the periphery of hematoma, and the hematoma is encapsulated and communicated with the lumen. Thus, the pseudoaneurysm is formed [13]. Extravascular TB was found in all patients of this group. TB adjacent to aneurysm includes miliary pulmonary TB, tuberculous spondylitis, pleural TB, renal TB, IPA or psoas abscess. Some patients have multiple TB sites. Other mechanisms of tuberculous aneurysm formation may include the following: mycobacterium TB reaching the vessel wall through vasa vasorum, spread of bacteria through lymphatic vessels around the artery, and direct implantation of bacteria on the internal surface of the vessel wall after vasculature trauma. Normal arterial intima is very resistant to infection. Atherosclerosis can alter the arterial lining and lower the resistance to infection [14]. At present, the incidence of TB is increasing in the elderly population who have the highest incidence of atherosclerosis; thus, it could be anticipated that seeding of the aorta would be a common finding.

CECT can provide valuable information about the morphology of AA, aortic wall enhancement, and the relationship between the aneurysm and adjacent tissue because of the higher quality spatial resolution. TBAA typically appears on CT as a focal, contrast-enhancing, saccular lumen, with an indistinct, irregular aortic wall [8]. Tuberculous aneurysms may occur anywhere along the arterial system [15] and usually occur as a solitary lesion [16]. The thoracic aorta is the most common location [17], because it is adjacent to the lungs and mediastinum where TB most commonly occurs. In this group, the incidence of TB pseudoaneurysm is the same in the thoracic or abdominal aorta [3]. Less frequently, femoral [18], iliac [19] and subclavian [12] arteries can also be affected. It has been reported that most of the aneurysms are saccular (98%) [2]. The shapes of TBAA in this study were all saccular, and nine of them were lobulated—the wall of the aneurysm was irregular. A lobulated aneurysm indicates more instability and higher risk of rupture. All aneurysms of the six patients who died were lobulated based on the saccular shape. The diameter of aneurysm ranged from 3 to 12 cm. The size of the aneurysm is neither a risk factor of rupture nor the necessity for influencing treatment [20]. Because one or more layers of mycotic aneurysm wall are missing, they all have the risk of rupture, no matter the size. However, the rapidly progressive growth of aneurysms (>5 mm in 2 weeks) is suggestive of an infectious etiology [8]. CT is the most sensitive imaging modality for the detection of calcification and gas bubbles. Calcification is reportedly very uncommon in TBAA [3]. Twelve patients in this group had calcification. The higher incidence of calcification in this study may be due to the older age of these 12 patients, with an average age of 68 years. Here, calcification was consistent with atherosclerosis. No patients showed gas bubbles, which may appear in and around aneurysm and could be indicative of high diagnostic reliability of bacterial infection [8]. Although gas bubble is an important sign of arterial infection, it is too rare to make the differential diagnosis. In this study, all cases of aneurysm ruptured at different locations of arterial wall, which was a process of forming pseudoaneurysms. In fact, three-tier structure of the aneurysm wall was incomplete, especially the sparse elastic fiber fracture of middle smooth muscle had broken. CT showed that the wall of some aneurysms was thin and there was no obvious soft tissue around it. Those lobulated, tension-free aneurysms are more likely to rupture, some of which look like a mess of mud and may have ruptured. CT can well display soft tissue and adjacent organ damage around tubercular aneurysm.

Eccentric periaortic surrounding soft tissue can show as a rim or septum enhancement by the administration of contrast material (venous phase) on CECT. Significant exudation around aneurysm was in three patients in this study. Exudation and edema around aneurysm suggest that the aneurysm was unstable and may have ruptured with extravasation. CT cannot differentiate between the exudation and edema from hematoma, whereas MR can provide more information due to its high tissue resolution. Lymph nodes adjacent to TBAA might also appear swollen and enhanced. These enlarged lymph nodes showed ring enhancement and necrosis in the center. TB can cause progressive enlargement of the surrounding lymph nodes, and the rupture of lymph nodes can spread to the adjacent aorta to form an aneurysm. Tuberculous aneurysm can also cause lymph node hyperplasia. A causal relationship between aneurysm and enlarged lymph nodes was not identified, especially in the late stage of the disease. IPA or psoas abscess is a common complication in the abdominal TBAA, presenting as a direct invasive infection with purulent materials occurring within the iliopsoas or psoas muscles. The typical features on CT are enlarged and swollen muscles with single or multiple relatively low-density areas and contrast enhanced rim of the abscess wall. In tuberculous spondylitis patients, TBAA can involve secondary spread from spine lesions. Primary and secondary pyogenic spondylitis manifests as erosion of the vertebral body and/or intervertebral disc on CT. Soft tissue swelling or abscess may be detected around the vertebral body. Pseudoaneurysm may develop adjacent to the eroded vertebral body, which greatly increases the risk of rupture during surgery. It was reported that an abdominal AA was iatrogenically ruptured during surgery for lumbar tuberculous spondylitis with psoas abscess [21]. In one case in this study, the patient’s AA was found during thoracic spine tuberculosis surgery. It is very important to evaluate the presence of aneurysm before surgery in patients with tuberculous spondylitis. Tuberculosis in other parts can usually be found in TBAA patients by CT scan, such as pulmonary TB, renal TB, and TB of the reproductive system.

Treatment of TBAA includes anti-TB chemotherapy treatment, open surgery (*in situ* reconstruction or extra-anatomic bypass), and endovascular treatment (embolization, aortic stent grafting) [22]. The mortality rate of tuberculous aneurysm is 35% in this group, which is still high. Early diagnosis and timely treatment are critical in reducing the mortality of TBAA. CT plays an important role in the diagnosis of TBAA, especially for some patients without conditions (economic reasons, no MRI/PET equipment), or with contraindications to MRI [3, 23]. TBAA shows saccular shapes on CT scans, which are imaged as unstressed. Another distinguishing feature is that the surrounding tissues and adjacent organs of TBAA are usually infected with TB, and most of them are accompanied by other sites of TB. Regular CT follow-up is also important for diagnosis. The limitation of this study was the absence of TB etiology in most cases with no follow-up in those who survived treatment. Future studies will address this limitation.

## 5. Conclusions

In conclusion, this study showed that TBAA typically appears on CT as a single saccular pseudoaneurysm, and the incidence is the same in the thoracic or abdominal aorta. Surrounding tissues and adjacent organs of pseudoaneurysm are infected with TB, and other sites of TB may be found.

## Data Availability

The datasets used during the current study are not publicly available due to strict requirements set out by the Human Ethics Research Committee regarding the storage and use of the data by authorised investigators.
